# A young girl with chronic isolated cervical lymphadenopathy found to have lupus lymphadenopathy, progressing to develop lupus nephritis: a case report

**DOI:** 10.1186/s13256-021-02949-5

**Published:** 2021-06-28

**Authors:** K. P. Jayawickreme, S. Subasinghe, S. Weerasinghe, L. Perera, P. Dissanayaka

**Affiliations:** Sri Jayawardenepura General Hospital, Sri Jayawardenepura Kotte, Sri Lanka

**Keywords:** Systemic lupus erythematosus, Lupus lymphadenopathy, Kikuchi–Fujimoto disease, Lymphoma

## Abstract

**Background:**

Systemic lupus erythematosus is a rare autoimmune disorder, with the prevalence in Asia ranging from 30 to 50/100,000. The diagnosis of systemic lupus erythematosus is made according to the 2019 European League Against Rheumatism/American College of Rheumatology classification criteria, and it does not contain lymphadenopathy as diagnostic criteria. However, lupus lymphadenopathy has an estimated prevalence of 5–7% at the onset of disease, and 12–15% at any stage of the disease.

**Case presentation:**

A 19-year-old Sinhalese girl had neck nodules since the age of 5 years, which increased in size and became tender since 1 year. She had alopecia and joint stiffness for 6 months. She presented with a 5-day history of worsening joint pain, fever, and painful, enlarging cervical nodules. She had tender cervical lymphadenopathy, and a vasculitic rash on both lower limbs. She had pancytopenia, an erythrocyte sedimentation rate of 92, positive antinuclear antibody titer, and high anti-double-stranded deoxyribonucleic acid (DNA), with low C3 and C4 complements. She had a high reticulocyte count of 5%, with direct and indirect antiglobulin tests being positive, indicating autoimmune hemolytic anemia. Lymph node biopsy showed moderate reactive follicular hyperplasia, with scattered plasma cells and immunoblasts, with varying degree of coagulative necrosis, suggestive of lupus lymphadenopathy. On immunohistochemistry of the lymph node biopsy, Bcl2 was negative, excluding lymphoma. Contrast-enhanced computed tomography of abdomen and chest was normal with no hepatosplenomegaly or lymphadenopathy. Skin biopsy showed leukocytoclastic vasculitis. Later, with development of generalized edema, she was found to have impaired renal function, and renal biopsy showed lupus nephritis. She was started on hydroxychloroquine, prednisolone, and mycophenolate mofetil, and her symptoms improved and lymphadenopathy regressed.

**Conclusion:**

In the case of cervical lymphadenopathy in a patient with systemic lupus erythematosus, the possibilities of lupus lymphadenopathy, Kikuchi–Fujimoto disease, and lymphoma should all be considered, after excluding secondary infection due to immunosuppression. Histology confirms the differentiation of these pathologies. It is important to differentiate the cause for lymphadenopathy in systemic lupus erythematosus as the outcome and treatment varies. Lupus lymphadenopathy is usually generalized, but isolated cervical lymphadenopathy could also rarely be the first presentation of systemic lupus erythematosus. Lupus lymphadenopathy can be the only presenting feature, and needs a high index in suspecting systemic lupus erythematosus, though it is not included in the diagnostic criteria.

## Background

Systemic lupus erythematosus (SLE) is a rare autoimmune disorder with antibodies against nuclear and cytoplasmic antigens causing multisystem involvement. The worldwide prevalence of SLE varies geographically, with the likely highest being in North America with a prevalence of 241/100,000, and in Asia ranging from 30 to 50/100,000 [1, 2]. The female-to-male ratio of SLE ranges from 2:1 to 15:1 among adults [1]. The currently recommended criteria in diagnosing SLE are the 2019 European League Against Rheumatism/American College of Rheumatology (EULAR/ACR) diagnostic criteria. Though these do not contain lymphadenopathy as a criterion, it can be a presenting feature, or the only presenting feature that should not be missed. The presence of lymphadenopathy in SLE can be multifactorial. Therefore, it is important to identify the etiology of lymphadenopathy in SLE, which could include lupus lymphadenitis, lymphoma associated with SLE, Kikuchi–Fujimoto disease, and secondary infection in an immunocompromised host, as the treatment and outcome may differ for each. Due to nonspecific presentations and multisystem involvement of SLE, it is important to have a high index of suspicion and prompt early diagnosis and treatment to improve survival.

## Case presentation

A 19-year-old Sinhalese girl had neck lumps since the age of 5 years, which were not evaluated before. They have now increased in size and become tender since 1 year. She had a rash on both her lower limbs for 6 years, but no other photosensitive rashes. She had alopecia for 4 years, and pain and morning stiffness of small and large joints for 6 months. She had no gritty eyes or visual impairment. She had loss of appetite but no loss of weight. She attained menarche at the age of 13 years, and had regular menstruation since then. She had no family history of rheumatological disease or malignancy. Her mother had a history of one miscarriage. She had no history of thrombotic phenomena. She presented with a 5-day history of worsening joint pain, fever, and painful, enlarging cervical nodules.

Her blood pressure was 110/70 mmHg and pulse rate was 80 beats per minute. She had bilateral soft tender cervical lymphadenopathy involving the anterior cervical region, and tender and swollen interphalangeal and carpometacarpal joints. She had an erythematous non-blanching macular rash suggestive of a vasculitic rash on both lower limbs, but no oral ulcers. She had no malar or photosensitive rashes and no digital ulcers or calcinosis cutis. She had no hepatosplenomegaly, with normal examination of the cardiovascular and respiratory systems. She had no focal neurological signs or proximal myopathy, and fundoscopy was normal.

She had pancytopenia (white blood cell count 2.3 × 10^6^, hemoglobin 10 g/dl, platelet count 118 × 10^6^). She had a high erythrocyte sedimentation rate (ESR) of 92, positive anti nuclear antibodies (ANA) titer of 1:640 on Hep-2 cells, and a high anti-double-stranded deoxyribonucleic acid (dsDNA) antibody level of 15 IU/ml (positive if > 10 IU/ml), with low C3 and C4 complements of 0.5 g/l (low < 0.785 g/l) and 0.08 g/l (low < 0.145 g/l), respectively, compatible with findings of systemic lupus erythematosus (SLE). Blood picture showed moderate mixed deficiency anemia with moderate rouleaux formation. She had mild iron deficiency anemia with hemoglobin of 9.5 g/dl, low serum ferritin of 8 μg/l, and low transferrin saturation of 15% with no bleeding manifestations. She had a high reticulocyte count of 5% (0.5–1.5%), with LDH of 182 U/l (140–280 U/l). Her direct and indirect antiglobulin tests were positive, and C3d specificity was positive with negative immunoglobulin (IgG) specificity indicating autoimmune hemolytic anemia. She was seen by a hematologist and was started on iron and folic acid supplements. Ultrasound scan of the neck showed reactive lymphadenopathy in the anterior cervical region. Lymph node biopsy showed moderate reactive follicular hyperplasia, with scattered plasma cells and immunoblasts, with varying degree of coagulative necrosis, suggestive of lupus lymphadenopathy. She did not have distorted nodular architecture with cortical and paracortical nodules with proliferation of histiocytes, and karyorrhexis, and crescentic histiocytic nuclei to suggest Kikuchi–Fujimoto disease. On immunohistochemistry of the lymph node biopsy, CD20 was positive in follicles, CD5 was positive in interfollicular areas, and Bcl2 was negative, excluding lymphoma histologically.

Ultrasound abdomen and contrast-enhanced computed tomography of abdomen and chest was normal with no hepatosplenomegaly or intraabdominal lymphadenopathy. Antibodies for cytomegalovirus, Epstein–Barr virus, human immunodeficiency virus (HIV), and Mantoux were negative. Skin biopsy showed fibrinoid necrosis of the vessels with fibrin extravasation suggestive of leukocytoclastic vasculitis. Her cytoplasmic anti-neutrophil cytoplasmic antibody (cANCA), perinuclear anti-neutrophil cytoplasmic antibody (pANCA), anti-Ro, anti-La, and antiphospholipid antibodies were negative, but anticardiolipin antibody was positive. However, she did not fit the criteria for antiphospholipid syndrome as she had no history of thrombotic phenomena or pregnancy-related complications of placental insufficiency. She was advised on using compression stockings during long distance travel, adequate hydration, and thromboprophylaxis during major surgery or during future pregnancies. Her serum creatinine was normal (77 μmol/l), urine protein-to-creatinine ratio was 343 mg/g, and she had no dysmorphic red cells in urine. She was seen by a nephrologist, and it was decided that a renal biopsy was not indicated at present. She was planned to be closely monitored, and to consider renal biopsy only if urine protein was more than 500 g/dl, or red cells in urine > 5–10/high power field (HPF) with > 10% dysmorphic red cells on follow-up. Ophthalmological evaluation showed no eye involvement of SLE.

Her SLE disease activity index (SLEDAI) at diagnosis was 22. She was seen by a rheumatologist, dermatologist, nephrologist, and hematologist for specialist opinion and was started on hydroxychloroquine 200 mg daily and prednisolone 30 mg daily along with gastric protection, and vitamin D and calcium supplements for bone protection. Her symptoms improved and lymphadenopathy regressed, and prednisolone was gradually tailed off over 3 months.

Six months after the initial presentation, she developed loss of appetite, generalized edema and was found to have a blood pressure of 135/100 mmHg, which later increased to 160/110 mmHg during the course. She was found to have an increased serum creatinine of 128 μmol/l, proteinuria with a urine-to-protein creatinine ratio (UPCR) of 3854 g/dl, and 20–25 red blood cells in urine on high-power field that were dysmorphic, with red cell urinary casts. Renal biopsy confirmed mixed grade III and grade V lupus nephritis, evidenced by less than 50% of the glomeruli showing proliferation without sclerosis, and diffuse thickening of the glomerular basement membrane on light microscopy, and subepithelial immune deposits on immunofluorescence. The SLEDAI during this flare was 26. She was treated with intravenous methylprednisolone 1 g daily for 3 days and intravenous cyclophosphamide pulse therapy with the first dose being 0.75 g/m^2^ followed by five doses of 0.5 g/m^2^ for induction. She was started on cilnidipine 10 mg twice per day and telmisartan 80 mg daily for blood pressure control and reduction of proteinuria, and mycophenolate mofetil 1.5 g twice per day for maintenance, and oral methylprednisolone 8 mg daily for lupus nephritis. Methylprednisolone was continued for 5 months and gradually tailed off, after which she was normotensive with UPCR less than 500 g/dl on several occasions, the latest being 125 g/dl 7 months after induction, with no hematuria on follow-up. The SLEDAI 7 months after induction for lupus nephritis was 2.

## Discussion

SLE is a multisystem disease, which is diagnosed by the presence of 10 or more of a total score of the diagnostic criteria and one or more clinical criteria for SLE according to the 2019 European League Against Rheumatism/American College of Rheumatology (EULAR/ACR) diagnostic criteria. This patient had arthritis, nonscarring alopecia, autoimmune hemolytic anemia, leukopenia, thrombocytopenia, subacute cutaneous lupus, positive ANA and dsDNA, anticardiolipin antibodies, and low C3 and C4, which account for the diagnosis of SLE with a score of 33 initially, and later with a score of 43 with the development of lupus nephritis, according to the 2019 EULAR/ACR diagnostic criteria for SLE [3]. Her earliest presentation and most significant complaint was cervical lymphadenopathy. However, lymphadenopathy is not among the diagnostic criteria of SLE [3].

Infective causes for lymphadenopathy such as cytomegalovirus, Epstein–Barr virus, HIV, and tuberculosis were initially considered, as they are a possibility in a patient on immunosuppressants. This is, however, unlikely in this case as this patient’s first presentation was lymphadenopathy that developed prior to starting any immunosuppressants. When investigating for lymphadenopathy in SLE, the possibilities of lymphoma associated with SLE, Kikuchi–Fujimoto disease (KFD) associated with SLE, and lupus lymphadenopathy (LL) should be considered [4]. Though they can have a similar presentation in SLE, differentiating these three conditions is important as their prognosis and mode of treatment are different.

LL has an estimated prevalence of 5–7% at onset of disease, and 12–15% at any stage of the disease [5]. This lymphadenopathy is typically soft, mobile, painful, and nonadherent to deep structures [5]. LL is typically generalized, and there are several case reports indicating that generalized lymphadenopathy can be the first manifestation of SLE [6–8]. This patient had isolated cervical lupus lymphadenopathy, which is a rare presentation, and there were no previous reported cases of isolated cervical LL as the first presentation in Sri Lanka. The presence of cervical lymphadenopathy since an early onset of 5 years of age is also a rare occurrence.

The histological findings of LL are usually nonspecific and consist of moderate follicular hyperplasia with increased vascularity, DNA deposits in vessel walls, and scattered immunoblasts and plasma cells. The presence of several degrees of coagulative necrosis with hematoxylin bodies is characteristic of LL, but is rarely seen. The histological appearance varies depending on the clinical manifestations and the disease course [4]. SLE disease activity measured by the British isles Lupus Assessment Group (BILAG) index has been found to be higher among patients with LL [9]. Some authors have reported a relationship between disease activity and LL, and have recommended that LL should be included among clinical findings indicating disease activity in SLE [9]. SLE patients with LL are found to show more constitutional symptoms such as fatigue, fever, weight loss, more cutaneous and mucosal signs including alopecia, higher incidence of hepatomegaly and splenomegaly, increased anti-dsDNA titers, and decreased complement levels. Irrespective of the presence of LL, there were no differences in renal or nervous system manifestations in SLE patients [9].

The possibility of a hematological malignancy such as lymphoma or chronic lymphocytic leukemia was excluded in this patient by immunohistochemistry of the lymph node biopsy, and did not have an indication for further evaluation with bone marrow biopsy. Several studies have reported an increased risk of malignancy, most significantly hematological malignancies such as lymphoma, in patients with autoimmune diseases like SLE [10]. Phenotypes of SLE that include hematological manifestations such as autoimmune hemolytic anemia, leukopenia, sicca syndrome with salivary gland enlargement, and pulmonary infiltrates are found to be associated with a higher risk of a lymphoma [11]. Since this patient had hematological manifestations as mentioned above, it was important to clearly exclude the possibility of a lymphoma. There is no relationship found between malignancy in SLE with antirheumatic cytotoxic drugs such as azathioprine and cyclophosphamide [11]. Another study showed that SLE in patients with younger age of onset, severe end organ damage, and longer disease duration was associated with non-Hodgkin’s lymphoma [12]. If the lymphadenopathy was due to lymphoma, it will not improve with antirheumatic drugs and steroids given in SLE, and will require specific cytotoxic chemotherapeutic agents. However, this patient’s lymphadenopathy regressed with treatment for SLE.

Kikuchi–Fujimoto disease (KFD), also known as histiocytic necrotizing lymphadenitis, has a very similar presentation to SLE, with female preponderance, young age of onset, and clinical features of fever, arthralgia, skin eruptions, lymphadenopathy, leukopenia, and elevated ESR as in this case [4]. The differentiating clinical feature is that KFD has an erythematous or ulcerating oropharyngitis, which is not typical of SLE [4]. Lymphadenopathy in KFD typically involves the cervical region, as in this case, but rarely causes generalized lymphadenopathy [13]. LL typically causes generalized lymphadenopathy, but this case had only isolated cervical lymphadenopathy and, thus, warranted active exclusion of KFD [6–8]. The diagnosis of KFD is by lymph node biopsy. Histology commonly shows geographic necrosis with foci of apoptotic cells with abundant karyorrhectic fragments surrounded by histiocytes [14]. Studies have shown occurrence of KFD prior to SLE in 30% of cases, simultaneous occurrence of both conditions in 47% of cases, and occurrence of KFD after diagnosis of SLE in 23% of cases [14]. KFD is self-limiting and resolves in 1–6 months. Such cases need to be assessed for SLE and followed up for a possible development of SLE subsequently [4, 14]. The coexistence of KFD in an SLE patient is associated with a more aggressive course, and treatment is needed to prevent relapse. KFD associated with SLE can be complicated with hemophagocytic syndrome, heart failure, or recurrent aseptic meningitis that requires intravenous immunoglobulin and corticosteroids [13]. Therefore, even in a patient diagnosed with LL, it is important to actively exclude concurrent KFD.

## Conclusion

When there is cervical lymphadenopathy in patient with SLE, the possibilities of LL, KFD, and lymphoma should all be considered after excluding infections due to immunosuppression,. Lymph node histology and immunohistochemistry differentiates these pathologies. LL is usually generalized, while isolated cervical lymphadenopathy is more common in KFD. SLE can precede, coincide with, and be followed by KFD. It is important to differentiate the cause for lymphadenopathy in SLE as the outcome and treatment varies. Isolated cervical lupus lymphadenopathy can be the only presenting feature, and needs a high index in suspecting SLE, though it is not included in the diagnostic criteria, as early diagnosis and treatment is important in improving outcome.

## Data Availability

Not applicable.

## Abbreviations

SLE: Systemic lupus erythematosus
LL: Lupus lymphadenopathy
KFD: Kikuchi–Fujimoto disease
ANA: Antinuclear antibodies
dsDNA: Double-stranded deoxyribonucleic acid
LDH: Lactate dehydrogenase
